# Field Root-Associated Microbiome Characteristics of *Astragalus membranaceus* and Its Transcriptomic Response to *Purpureocillium lilacinum* BP2-7 Treatment

**DOI:** 10.3390/jof12040243

**Published:** 2026-03-26

**Authors:** Haiping Jiang, Hujun Xu, Zhaoyun Meng, Ke Hao, Yuze Yang, Yujia Zhao, Qingzhi Yao, Min Li

**Affiliations:** 1 College of Life Science and Technology, Inner Mongolia Normal University, Huhhot 010022, China; jianghaiping7@163.com (H.J.); xhj15202585996@163.com (H.X.); 15204984933@163.com (K.H.); 20244015042@mails.imnu.edu.cn (Y.Y.); 20201102349@mails.imnu.edu.cn (Y.Z.); 2 Key Laboratory of Biodiversity Conservation and Sustainable Utilization for College and University of Inner Mongolia Autonomous Region, Hohhot 010022, China; 3 Ulanqab Center for Disease Control and Prevention, Ulanqab 012000, China; mzy126825@163.com; 4 College of Grassland Science, Inner Mongolia Agricultural University, Hohhot 010011, China; yaoqingzhi@163.com

**Keywords:** *Astragalus membranaceus*, root rot, biocontrol fungi, transcriptome sequencing, *Purpureocillium lilacinus*

## Abstract

*Astragalus membranaceus* suffers severe yield and quality losses due to root rot caused by *Fusarium solani*. To address this, we analyzed the root-associated microbial communities of healthy and diseased plants in northwest China using high-throughput sequencing. Combining community analysis with pot experiments and transcriptomic profiling, we elucidated the molecular mechanisms by which the biocontrol fungus *Purpureocilliu lilacinum* BP2-7 suppresses root rot. Root rot reshaped root-associated microbial structure, affecting fungal diversity more than bacterial diversity. The antagonistic effect of *P. lilacinum* BP2-7 against *F. solani* reached 71.43% in plate assays and 63.7% control efficacy in pot experiments, representing the first report of *P. lilacinum* application for managing root rot in *A. membranaceus*. Transcriptomic analysis revealed that *P. lilacinum* BP2-7 promotes the transition of plants from a damaged to a recovering state by modulating translation and metabolic processes, and enhancing protein homeostasis, while moderately downregulating defense-related responses to alleviate pathogen-induced excessive defense mechanisms. Additionally, twenty candidate genes involved in the direct inhibition of *F. solani* were identified, suggesting a role in enhancing host resistance. This study supports eco-friendly biocontrol strategies and advances understanding of plant–microbe interactions for managing soil-borne diseases in other important crops.

## 1. Introduction

*Astragalus membranaceus* (milk vetch; known in traditional Chinese medicine as Huangqi or Huang Chi, where it has a long history of medicinal use) is a perennial herbaceous plant, and its roots are the primary medicinal part. They are rich in polysaccharides, saponins, and flavonoids, and exhibit diverse biological activities, including immunomodulatory and antitumor effects, as well as therapeutic potential against diabetes and neurological diseases, metabolic regulation, and protective effects on the liver, kidneys, and intestine [1,2]. With the continuous increase in market demand, large-scale cultivation and continuous monocropping of *A. membranaceus* have rapidly expanded. However, unfavorable soil conditions and monoculture practices promote the accumulation of soil-borne pathogens. Consequently, *A. membranaceus* is prone to root rot, mainly caused by *Fusarium* species such as *F*. *solani* and *F. oxysporum* [3,4]. Typical symptoms include blackening, rotting, and softening of the roots, which further lead to plant wilting, chlorosis, growth inhibition, and even large-scale plant death, resulting in severe yield losses and reduced ecosystem stability [5]. At present, the management of root rot relies primarily on agronomic practices and chemical control methods. However, agronomic measures such as intercropping and crop rotation are constrained by land availability and time conditions and often reduce economic benefits [6]. Chemical control, on the other hand, may cause pesticide residues, environmental pollution, and ecological imbalance [7]. In contrast, biological control, as an environmentally friendly and sustainable alternative strategy, can effectively suppress disease occurrence while ensuring ecological safety. Root-associated microbial communities play important roles in plant growth and stress responses. Field studies in crops such as tobacco, banana, and sugar beet have shown that plants with different health statuses are often accompanied by significant differences in the structure of root-associated microbial communities [8,9,10]. Such field investigations provide important information for understanding the ecological associations between plants and microorganisms. However, due to the high complexity and heterogeneity of field soil environments, field surveys alone make it difficult to directly attribute plant health status to specific microbial taxa or functions. To complement field observations, experiments under controlled conditions are commonly used to investigate the potential functions of microorganisms and plant physiological responses in simplified and reproducible environments. Previous studies have demonstrated that various microbial agents can effectively suppress soil-borne pathogens under greenhouse or laboratory conditions. For example, the myxobacterium *Corallococcus* isolate EGB achieved a control efficacy of 79.6% against cucumber *Fusarium* wilt under greenhouse conditions [11]. Similarly, *Bacillus velezensis* YW17 isolated from forest rhizosphere soil effectively inhibited *F*. *oxysporum*, the causal agent of ginseng root rot [12]. In addition, cross-kingdom synthetic microbial communities (SynComs) have also shown strong control effects against tomato *Fusarium* wilt [13]. These studies highlight the potential of microbial biocontrol agents in managing soil-borne diseases and provide valuable references for developing strategies to control root rot in *A. membranaceus*.

However, reports on biocontrol microorganisms against *A. membranaceus* root rot remain limited, and the documented taxa mainly include *Stenotrophomonas*, *Rhizobium*, and *Ochrobactrum* [4]. In addition, *Serratia premortalis* KRS006 has been reported as an effective biocontrol agent against *A. membranaceus* root rot, exhibiting inhibition rates of 42–55% against multiple pathogenic *Fusarium* isolates [14]. *B*. *paralicheniformis* 2–12 can disrupt the hyphal structure of *F. oxysporum* by releasing antifungal volatile organic compounds (VOCs), reducing the incidence of *Astragalus* root rot from 100% to 13.33% [15]. Nevertheless, the diversity of currently available biocontrol resources remains low, which limits their ability to cope with pathogen variation and environmental fluctuations, resulting in insufficient stability of control efficacy. Therefore, for *A. membranaceus* root rot, it is urgent to explore novel, diverse, and environmentally adaptable biocontrol microbial resources to overcome the limitations of existing management strategies and address the increasing severity of soil-borne diseases against the background of global climate change.

*Purpureocillium lilacinum* (formerly *Paecilomyces lilacinus*) is a widely distributed and easily culturable fungus. Previous studies have demonstrated that this species possesses broad-spectrum biocontrol activity and plant growth-promoting functions. It has been reported that *P. lilacinum* effectively suppresses a wide range of pests and phytopathogenic fungi, such as the root-knot nematode *Meloidogyne* [16], *Fusarium* [17], and *Verticillium dahliae* [18]. Moreover, *P. lilacinum* can promote plant growth through multiple mechanisms, including the production of indole-3-acetic acid (IAA), stimulation of root development [19], and enhancement of phosphorus solubilization and utilization [20]. Therefore, *P. lilacinum* is considered a beneficial microorganism with both biocontrol and plant growth-promoting properties. These beneficial effects have been validated in crops such as eggplant [19], maize [21], and wheat [17]. However, the application of *P. lilacinum* in medicinal plants, particularly in the control of *A. membranaceus* root rot, has not yet been reported. Given the limited availability of current biocontrol approaches, *P. lilacinum* BP2-7 shows promising application potential, but the molecular interaction mechanisms among the host, pathogen, and biocontrol fungus remain unclear.

Transcriptome sequencing is a high-throughput sequencing-based analytical approach that enables systematic comparisons of differential gene expression under different treatments or conditions, and it has been widely applied in studies of plant responses to biotic stresses. With broad coverage and high data resolution, this technique provides fundamental data support for analyzing transcriptional changes in plants under specific conditions [22]. For example, time-series transcriptome analysis of *Arabidopsis thaliana* infected by *Botrytis cinerea* revealed temporal changes in the expression levels of genes associated with cell wall-related metabolism, redox processes, and multiple phytohormone-related pathways, thereby describing the dynamic transcriptional features of plants under pathogen stress [23]. In addition, RNA-seq analysis of the *F. graminearum* ΔFgDUG3 mutant showed differences in gene expression profiles across multiple functional categories related to metabolism and signaling, indicating that transcriptome analysis can serve as an effective tool for comparing expression characteristics among different genetic backgrounds [24].

The aim of this study was to characterize the root-associated microbial communities of *A*. *membranaceus* under different health conditions and to analyze host transcriptomic responses to *P*. *lilacinum* BP2-7 treatment under *F*. *solani* stress.

## 2. Materials and Methods

### 2.1. Field Sample Collection

The research area is located in Kunduitan Village (KDT) (110°48′00″ E, 41°26′24″ N, altitude 1569.5 m) and Shuilongtan Village (SLT) (110°44′24″ E, 41°28′48″ N, altitude 1498.5 m), Darhan Muminggan Union Banner, Baotou City, Inner Mongolia, China. The sampling sites have been cultivated with *A*. *membranaceus* for a long time under agricultural management practices. Roots were collected from two-year-old healthy and diseased *A. membranaceus* plants. The roots were thoroughly washed and surface-sterilized with 70% ethanol for 40 s followed by 4% sodium hypochlorite for 3 min, and then rinsed thoroughly with sterile water [25]. The samples were sent to Biomarker Biotechnology Co., Ltd. (Qingdao, China) for high-throughput microbial diversity sequencing. Meanwhile, the rhizosphere soil samples were air-dried and sieved through a 2-mm mesh for subsequent analysis.

### 2.2. Root-Associated Microbial Community Sequencing and Analysis

Total DNA was extracted from the samples using the TGuide S96 magnetic bead-based DNA extraction kit, and nucleic acid concentrations were measured with a microplate reader. PCR amplification was performed targeting the bacterial 16S rRNA gene V3–V4 region and the fungal ITS1 region. The bacterial primers were 338F (5′-ACTCCTACGGGAGGCAGCA-3′) and 806R (5′-GGACTACHVGGGTWTCTAAT-3′), and the fungal primers were ITS1 (5′-CTTGGTCATTTAGAGGAAGTAA-3′) and ITS2 (5′-GCTGCGTTCTTCATCGATGC-3′) [26,27]. PCR reactions were carried out in a 10 μL volume containing 50 ng genomic DNA, 5 μL KOD FX Neo Buffer, 0.2 μL KOD FX Neo, 2 μL dNTPs (2 mM each), 0.3 μL of each primer, and ddH_2_O to the final volume. The PCR program was as follows: initial denaturation at 95 °C for 5 min; 25 cycles of 95 °C for 30 s, 50 °C for 30 s, and 72 °C for 40 s, followed by a final extension at 72 °C for 7 min. PCR products were examined by 1.8% agarose gel electrophoresis, excised, and purified using the Omega DNA purification kit (Omega Bio-tek, Norcross, GA, USA). Purified amplicons were quality-checked using the Qsep-400 system (BiOptic, Inc., New Taipei City, Taiwan, ROC). Libraries with a concentration of 2 nM were sequenced on the Illumina NovaSeq 6000 platform (Illumina, San Diego, CA, USA).

### 2.3. Analysis of Soil Physicochemical Properties

Soil pH was determined using a 2.5:1 ratio of soil to deionized water, followed by shaking to homogenize, standing for 30 min, and measurement with a pH meter. Soil organic matter (SOM) was measured using the potassium dichromate volumetric method. Soil available potassium (AK) was extracted with ammonium acetate and determined by flame photometry. Soil available phosphorus (AP) was extracted with 0.5 mol/L NaHCO_3_ and quantified by the molybdenum–antimony colorimetric method. Soil available sulfur (AS) was extracted with phosphate solution and determined using the barium sulfate turbidimetric method. Soil available copper (ACu) was extracted with DTPA-TEA and measured by spectrophotometry. Soil available chlorine (ACl) was determined by nitrate titration. Soil nitrate nitrogen (NN) was quantified using the phenoldisulfonic acid method, and soil ammonium nitrogen (AN) was determined by the indophenol blue spectrophotometric method [28].

### 2.4. Isolation and Identification of Fungal Isolates

The pathogenic fungus *F*. *solani* was previously isolated from *A*. *membranaceus* plants showing root rot symptoms and preserved in our laboratory. The pathogenicity of the isolate was verified according to Koch’s postulates. Healthy *A. membranaceus* root segments were inoculated with mycelial plugs of the isolate and incubated under sterile conditions. Typical root rot symptoms developed on the inoculated tissues, whereas no symptoms were observed in the control. The pathogen was re-isolated from the infected tissues and identified as *F*. *solani*.

A fungal isolate identified as *P*. *lilacinum* was isolated from healthy three-year-old *A. membranaceus* roots collected in Yinhao Town, Guyang County, Baotou City, Inner Mongolia, China (110°18′30″ E, 41°6′7″ N, altitude 1515.52 m).

**Figure A1:**
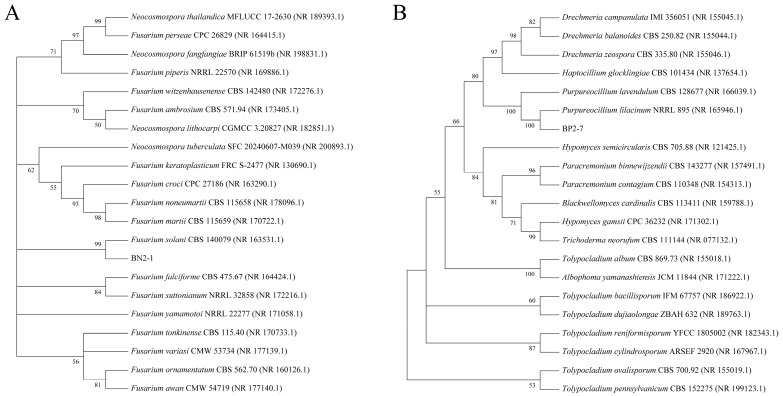
Phylogenetic trees were constructed using the neighbor-joining (NJ) method based on ITS sequences amplified with primers ITS1 and ITS4. Numbers at the branch nodes indicate bootstrap support values from 1000 replicates. (**A**): BN2-2; (**B**): BP2-7.

Genomic DNA was extracted using the resin-based method. The biocontrol fungus was amplified using the universal fungal primers ITS1 and ITS4 [27]. The PCR products were purified and subsequently subjected to bidirectional sequencing by Qingke Biotechnology Co., Ltd. (Beijing, China) (Table A1 and Table A2). Phylogenetic analysis based on ITS sequences was performed using the neighbor-joining method in MEGA 12.1 software with 1000 bootstrap replicates. The phylogenetic tree is shown in Appendix A Figure A1.

### 2.5. In Vitro Antagonistic Activity Against F. solani

In vitro antagonistic activity against *F. solani* was evaluated using a plate confrontation assay, as described by Skidmore and Dickinson [29] with slight modifications. A 6-mm diameter mycelial plug of *F. solani* was placed at the center of a PDA plate. Four mycelial plugs (6 mm in diameter) of the antagonistic strain were inoculated at four equidistant points 2.5 cm from the center of the plate. Plates inoculated only with *F. solani* served as the control. All plates were incubated at 25 °C until the control colony nearly covered the plate. The experiment was performed in triplicate, and the results are expressed as mean ± standard deviation (SD). Colony diameters were measured, and inhibition rates were calculated using the following formula.

Inhibition rate (%) = [(Colony diameter of control − Colony diameter of treatment)/Colony diameter of control] × 100

### 2.6. Greenhouse Experiment on A. membranaceus Seedlings

Seeds of *A. membranaceus* were surface sterilized by soaking in NaClO solution (available chlorine ≥ 10%) for 20 min, and then placed in sterile Petri dishes containing moistened filter paper for germination in a growth chamber (16 h light/8 h dark). After cotyledons emerged, the seedlings were transplanted into sterilized bags filled with 1.5 L of substrate (soil:vermiculite = 2:1), in which the soil was prepared by mixing KDT and SLT soil samples at a 1:1 ratio (*v*/*v*), with four seedlings per bag. When the first true leaf developed, seedlings of consistent growth were selected for experiments.

Experimental setup with 4 treatment groups—sterile water control group (CK), pathogen treatment (F), biocontrol fungus treatment group (P) and combined pathogen and *P. lilacinum* BP2-7 treatment (FP)—with ten replicates per treatment. The activated pathogen and *P. lilacinum* BP2-7 were separately inoculated into PDB and incubated on a rotary shaker at 28 °C and 180 r/min for 7 days. The fungal suspensions were filtered through four layers of sterile gauze to remove mycelial fragments. The filtrates were then centrifuged at 4500 rpm for 15 min, and the supernatants were discarded. The resulting spore pellets were resuspended in sterile water, and spore concentrations were determined using a hemocytometer. Finally, the spore suspensions were diluted with sterile water to a final concentration of 1 × 10^6^ cfu/mL for subsequent experiments. The roots of *A. membranaceus* seedlings were wounded with sterile blades. In the F group, 50 mL of *F. solani* spore suspension was applied to the wounded roots. The CK group received 50 mL of sterile water. In the P group, 50 mL of the *P. lilacinum* BP2-7 spore suspension was applied to the wounded roots. In the FP group, seedlings were first inoculated with 50 mL of the *P. lilacinum* BP2-7 spore suspension, followed by inoculation with 50 mL of *F. solani* spore suspension after 7 days. During the seedling growth period, plants were irrigated with 200 mL water every three days. After 45 days, all samples were collected. Roots were washed with physiological saline to remove surface debris and blotted dry with filter paper. Scissors were decontaminated with RNaseZap before excising the roots, which were immediately placed into cryogenic tubes (kept partially submerged in liquid nitrogen) and stored at −80 °C. Each 2 g portion of root tissue was treated as one biological replicate. Three biological replicates were included for each treatment group, resulting in nine samples in total.

This study modified the methods of Vestberg, M. [30] and Xue, L. et al. [31] according to our specific experimental conditions. The severity of root rot in *A. membranaceus* was assessed and classified into six grades (Table A3). The calculation formulas for each index were as follows:

Disease incidence (%) = (Number of symptomatic plants/Total number of investigated plants) × 100

Disease severity index (%) = [Σ (Severity grade × Number of plants at that grade)]/(Maximum severity grade × Total number of plants) × 100

Relative disease severity reduction (%) = (Disease severity index of control − Disease severity index of treatment)/Disease severity index of control × 100

### 2.7. RNA Sequencing of A. membranaceus

Root samples of *A. membranaceus* seedlings from different treatments were submitted to Majorbio Bio-Pharm Technology Co., Ltd. (Shanghai, China) for de novo transcriptome sequencing. Total RNA was extracted using the MJZol Total RNA Extraction Kit (Shanghai Majorbio Bio-pharm Biotechnology Co., Ltd., Shanghai, China), and RNA concentration and purity were assessed with a Nanodrop 2000 spectrophotometer (Thermo Fisher Scientific, Waltham, MA, USA). mRNA was enriched using Oligo dT primers included in the Illumina Stranded mRNA Prep, Ligation kit (Illumina, San Diego, CA, USA) were used for library construction. and randomly fragmented with fragmentation buffer to facilitate subsequent sequencing. Under the action of reverse transcriptase, mRNA was reverse-transcribed into cDNA, and sticky ends were converted to blunt ends using End Repair Mix. The ligated fragments were then purified, library-enriched, and sequenced on the Illumina NovaSeq 6000 platform. Raw sequencing data were filtered using Fastp 0.19.5 software to remove adaptor sequences, low-quality reads, reads with a high proportion of ambiguous bases, and overly short reads, resulting in high-quality clean data. Since no reference genome was available in this study, de novo assembly was performed using Trinity 2.8.5, and the assembled sequences were further optimized, filtered, and evaluated with TransRate 1.0.3 and BUSCO 3.0.2.

### 2.8. Statistical Analysis

Raw sequencing data were quality-filtered using Trimmomatic v0.33, and primer sequences were removed with cutadapt 1.9.1 to obtain high-quality clean reads. Sequence denoising was performed with the DADA2 pipeline implemented in QIIME2 2020.6 [32,33], which included merging of paired-end reads and removal of chimeras, yielding the final effective dataset. The resulting sequences were clustered into operational taxonomic units (OTUs) at a 97% similarity threshold using USEARCH v10.0, and the most abundant representative sequence of each OTU was subjected to taxonomic classification. Chord diagrams were generated with the “circlize” package in R 4.3.2, and alpha-diversity indices (Shannon, Chao1, Simpson, and ACE) were calculated using SPSS 27 software. Principal coordinate analysis (PCoA) based on Bray–Curtis and Jaccard distance metrics was conducted to assess beta-diversity. Mantel tests were performed using the “ggcor 0.9.8.1” package in R, while network topological features were computed with the “igraph 2.1.4” package. Strongly correlated edges were identified based on correlation coefficients and other criteria.

For transcriptomic analysis, differentially expressed genes (DEGs) were identified with the thresholds false discovery rate (FDR) < 0.05 and |log2 fold change| ≥ 1. Gene Ontology (GO) enrichment bar plots were generated using the “ggplot2” package in R, and Kyoto Encyclopedia of Genes and Genomes (KEGG) bubble plots were created using the “clusterProfiler 3.18” and “ggplot2” packages.

## 3. Results

### 3.1. Root Rot Reshapes the Root-Associated Microbial Community of A. membranaceus

**Figure 1:**
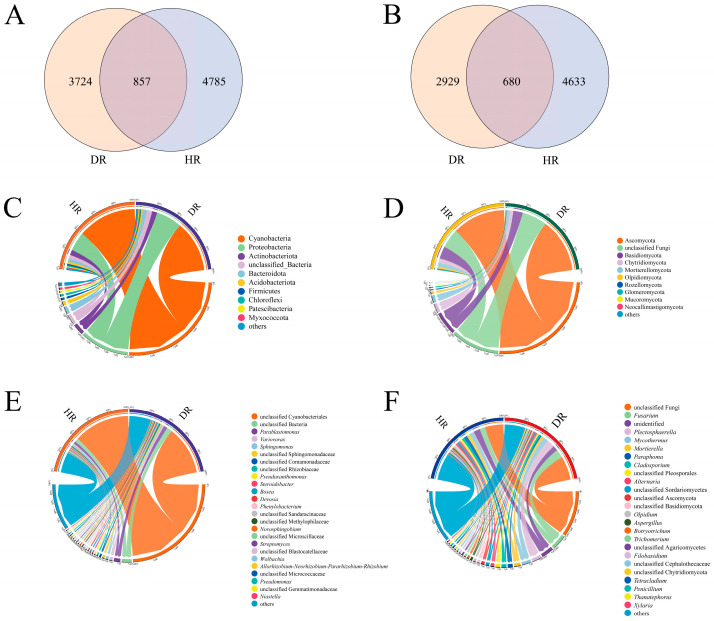
Microbial taxonomic composition and distribution in field-collected healthy and diseased *A. membranaceus* roots. (**A**) Venn diagram of bacterial OTUs; (**B**) Venn diagram of fungal OTUs; (**C**) chord diagram of bacterial phyla; (**D**) chord diagram of fungal phyla; (**E**) chord diagram of bacterial genera; and (**F**) chord diagram of fungal genera. HR, healthy roots; DR, diseased roots.

High-throughput sequencing analysis of microbial communities in healthy and diseased *A. membranaceus* roots (Figure 1A,B) revealed that the number of bacterial and fungal operational taxonomic units (OTUs) in the healthy and diseased roots were 5642/4581 and 5313/3609, respectively. The number of bacterial and fungal OTUs in the diseased roots was lower than in the healthy roots, indicating that the disease reduced the microbial OTU diversity in the roots of *A*. *membranaceus*. OTUs with a relative abundance greater than 10% were defined as dominant phyla, and those with a relative abundance greater than 1% were defined as dominant genera. In the healthy and diseased *A*. *membranaceus* roots (Figure 1C–F), the dominant bacterial phyla were Cyanobacteria (59.85%/52.00%) and Proteobacteria (19.30%/26.26%), while the dominant bacterial genera were *Parablastomonas* (2.54%/3.78%), *Variovorax* (1.13%/1.82%), and *Sphingomonas* (1.33%/1.02%). The dominant fungal phyla in the healthy roots were Ascomycota (55.03%) and Basidiomycota (12.54%), whereas in the diseased roots, only Ascomycota (56.08%) was the dominant fungal phylum. The dominant fungal genera in the healthy roots were *Fusarium* (6.19%), *Mycothermus* (4.20%), *Mortierella* (3.70%), *Paraphoma* (3.47%), *Cladosporium* (2.90%), *Alternaria* (2.19%), *Olpidium* (2.09%), *Botryotrichum* (1.40%), and *Aspergillus* (1.33%). After disease onset, the relative abundances of *Aspergillus* (0.96%), *Botryotrichum* (0.77%) and *Olpidium* (0.34%) decreased and were no longer dominant genera. *Plectosphaerella* (8.93%) and *Thanatephorus* (1.07%) increased in relative abundance and became dominant genera. Compared with healthy roots, most dominant fungal phyla and genera in diseased roots showed reduced relative abundance, whereas the relative abundances of *Fusarium* (9.20%), *Plectosphaerella* (8.93%), *Mycothermus* (5.32%), and *Thanatephorus* (1.07%) increased. These findings suggest that the occurrence of root rot significantly altered the microbial community structure and the composition of dominant groups in the roots of *A*. *membranaceus*, with the fungal community exhibiting stronger community reconstruction characteristics.

### 3.2. Differential Responses of Bacterial and Fungal Communities to Root Rot

**Figure 2:**
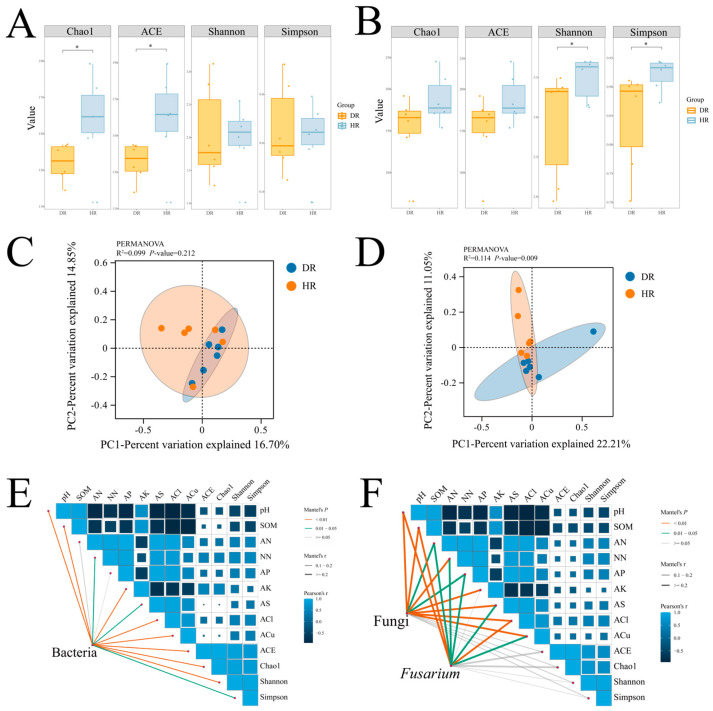
Microbial community analyses were performed on samples obtained from field-collected healthy and diseased *A. membranaceus* roots and rhizosphere soil. (**A**) Box plot of Alpha diversity analysis of bacterial communities at the order level; (**B**) box plot of Alpha diversity analysis of fungal communities at the family level; (**C**) PCoA analysis of bacterial communities; (**D**) PCoA analysis of fungal communities; (**E**) Mantel test for evaluating the correlation between core bacterial communities and environmental factors; (**F**) correlation between core fungal communities and environmental factors. HR, healthy roots; DR, diseased roots. Alpha diversity was analyzed using a one-tailed test. * indicates a significant difference at *p* < 0.05.

Microbial community diversity was evaluated using the Simpson and Shannon indices, while community richness was assessed with the ACE and Chao1 indices. Alpha-diversity analysis of the root-associated microbial communities in healthy and diseased *A. membranaceus* roots (Figure 2A,B) showed that, in the bacterial community, the ACE and Chao1 indices of healthy roots were significantly higher than those of diseased roots, indicating that healthy plants possessed greater bacterial richness. In contrast, within the fungal community, the Simpson and Shannon indices of healthy roots were significantly higher than those of diseased roots. In addition, Beta-diversity analysis based on the binary Jaccard distance (Figure 2C,D) revealed that fungal community structures of healthy and diseased roots differed significantly (PERMANOVA, *p* < 0.05), whereas the bacterial community structures showed no significant difference (PERMANOVA, *p* > 0.05), indicating that fungal communities were more responsive to root rot than bacterial communities.

To elucidate how inherent soil conditions influence the structure of bacterial and fungal communities, we further analyzed the correlations between the bacteria and fungi in *A. membranaceus* roots and the soil physicochemical factors (Figure 2E,F). SOM and pH are significantly negatively correlated with the diversity and abundance of bacteria and fungi, while other physicochemical factors generally show positive correlations. Specifically, the bacterial community is significantly positively correlated with AK, ACL, and ACu; the fungal community is significantly positively correlated with NN, AK, AS, ACL, and ACu.

### 3.3. Root Rot Infection Altered the Topological Structure of Root-Associated Microbial Interaction Networks

**Figure 3:**
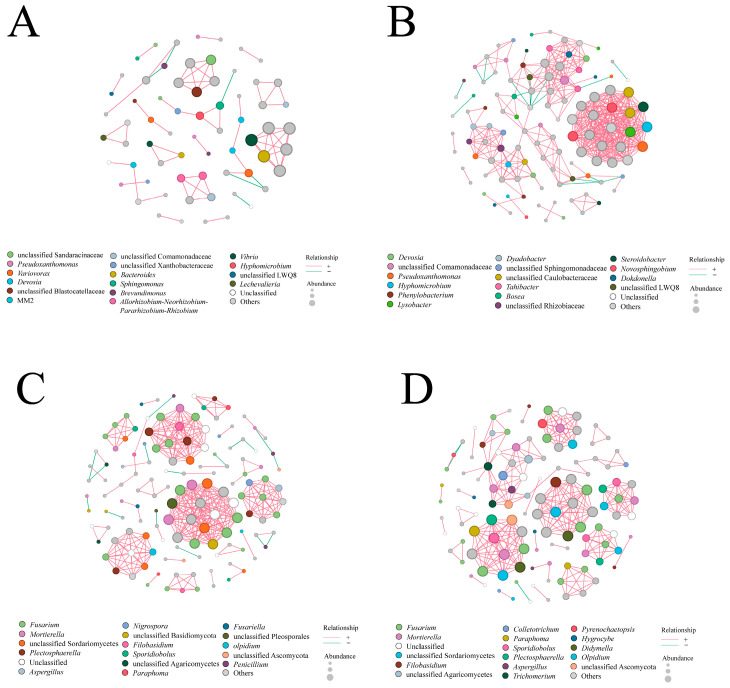
Co-occurrence network analysis of bacterial and fungal communities in field-collected healthy and diseased *A. membranaceus* roots. (**A**) Genus-level co-occurrence network of bacterial communities in healthy roots; (**B**) genus-level co-occurrence network of bacterial communities in diseased roots; (**C**) genus-level co-occurrence network of fungal communities in healthy roots; (**D**) genus-level co-occurrence network of fungal communities in diseased roots.

Co-occurrence network analysis of bacterial and fungal communities in healthy and diseased *A. membranaceus* roots (Figure 3A–D), conducted under the filtering criteria of relative abundance ≥ 0.005, |r| ≥ 0.6, and *p* < 0.05, revealed pronounced structural differences between networks before and after disease onset. In diseased roots, the total number of edges in the bacterial network increased by 508.33%, and the average degree increased by 252.10%. In contrast, the fungal network showed a 14.20% decrease in total edges and a 4.97% decrease in average degree. These results indicate that the bacterial and fungal communities within plant roots are significantly restructured following the development of root rot.

### 3.4. P. lilacinum Effectively Inhibits F. solani Infection and Promotes Growth of A. membranaceus

**Figure 4:**
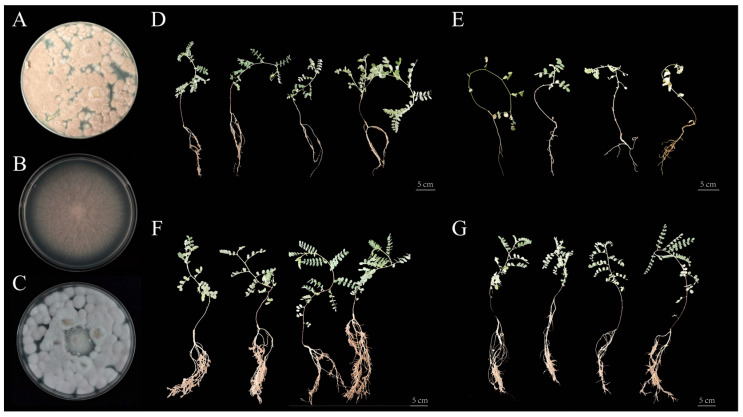
(**A**) Isolated *P. lilacinum* BP2-7; (**B**) control with single inoculation of *F. solani* BN2-2; (**C**) plate confrontation assay between *P. lilacinum* BP2-7 (inoculated around the perimeter of the medium) and *F. solani* BN2-2 (inoculated at the center of the medium); (**D**) growth status of *A. membranaceus* in the control group; (**E**) growth status of *A. membranaceus* in the pathogen-treated group; (**F**) growth status of *A. membranaceus* in the biocontrol fungus treatment group; (**G**) growth status of *A. membranaceus* in the dual-treatment group with the pathogen and biocontrol fungus.

The results of the plate confrontation assay showed that it inhibited the pathogen *F. solani* BN2-2 with an inhibition rate of 71.43 ± 1.81% (Figure 4B,C).

The results of the pot experiment showed that, compared with the control (Figure 4D), seedlings in the pathogen-treated group exhibited stunted growth, markedly reduced lateral roots, brown leaf spots, massive leaf abscission, and eventual plant death, consistent with the symptoms observed in the collected *A. membranaceus* plants showing root rot (Figure 4E). In contrast, seedlings in the pathogen–biocontrol dual-treatment group (hereafter referred to as the dual-treatment group) displayed markedly reduced pathogen damage, with growth phenotypes nearly indistinguishable from the uninfected control (Figure 4G). The control efficacy of the biocontrol fungus against the pathogen was calculated to be 63.7%. The disease assessment results are summarized in Appendix A Table A4. The biocontrol fungus treatment group showed good growth performance (Figure 4F).

### 3.5. P. lilacinus BP2-7 Primarily Controls F. solani Through the Positive Regulation of Genes

**Figure 5:**
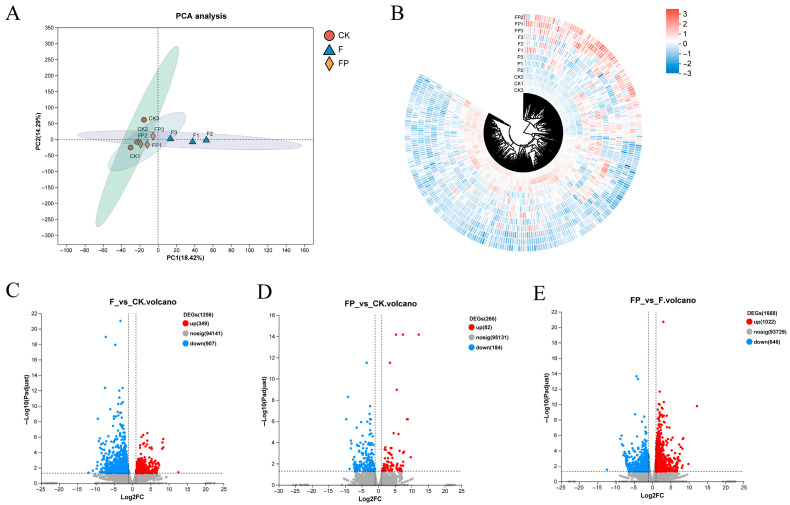
Statistics of DEGs among different treatment groups: (**A**) Scatter plot of the first and second principal components (PC1 and PC2) from PCA of different treatment groups; (**B**) circular heatmap of DEGs; (**C**) volcano plot of DEGs between the dual-treatment group and the control group; (**D**) volcano plot of DEGs between the pathogen-treated group and the control group; (**E**) volcano plot of DEGs between the dual-treatment group and the pathogen-treated group. CK, control group; F, pathogen-treated group; FP, dual-treatment group; DEGs, differentially expressed genes.

To further evaluate differences in expression patterns among the treatment groups, Principal Component Analysis (PCA) based on transcripts per million (TPM) was performed (Figure 5A). Samples within each treatment clustered closely, while samples between treatments were well separated, indicating good reproducibility and significant treatment effects. Hierarchical clustering of DEGs was visualized via a heatmap (Figure 5B), revealing numerous genes with opposite expression trends between the dual-treatment group and the control; genes significantly upregulated in the dual-treatment group were generally expressed at low levels in the control.

**Figure 6:**
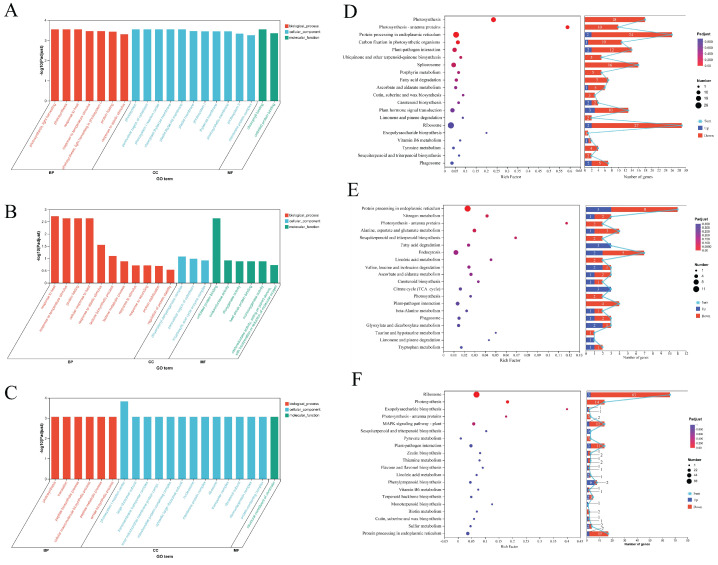
(**A**) Enrichment analysis of DEGs between the pathogen-treated group and the control group; (**B**) enrichment analysis of DEGs between the dual-treatment group and the control group; (**C**) enrichment analysis of DEGs between the dual-treatment group and the pathogen-treated group; (**D**) KEGG enrichment analysis of the pathogen-treated group versus the control group; (**E**) KEGG enrichment analysis of the dual-treatment group versus the control group; (**F**) KEGG enrichment analysis of the dual-treatment group versus the pathogen-treated group. GO, Gene Ontology; BP, biological process; CC, cellular component; MF, molecular function; DEGs, differentially expressed genes; KEGG, Kyoto Encyclopedia of Genes and Genomes.

To more clearly understand how *P. lilacinum* BP2-7 influences the *A. membranaceus* response to *F. solani*, we analyzed the volcano plots of DEGs from the *A. membranaceus* under different treatments (Figure 6C–E). The results showed that 1256 DEGs were detected between the pathogen-treated group and the control group, with significantly more downregulated genes (907) than upregulated genes (349), indicating that pathogen infection notably disrupted the host gene expression network, primarily through negative regulation, which inhibited the host’s physiological and metabolic activities. In contrast, only 266 DEGs (82 upregulated and 184 downregulated) were identified between the dual treatment group and the control group, with a much weaker differential expression, suggesting that the intervention of the biocontrol fungus alleviated the transcriptional imbalance caused by the pathogen, bringing the host’s expression pattern closer to a normal state. A further comparison between the dual treatment group and the pathogen-treated group identified 1668 DEGs, with upregulated genes (1022) significantly outnumbering downregulated genes (646), suggesting that the biocontrol fungus primarily exerts its protective effect against *Astragalus* root rot disease through positive regulation of genes.

### 3.6. Functional Enrichment Analysis of A. membranaceus DEGs in Response to P. lilacinum and F. solani

To systematically characterize the biological functions of the DEGs, this study conducted GO functional enrichment and KEGG pathway enrichment analyses on three sets of DEGs. In the DEGs between the control group and the pathogen-treated group (Figure 6A,D), the GO and KEGG enrichment analyses indicated that pathogen infection markedly reshaped the physiological and metabolic processes of the plant. Photosynthesis-related GO terms, such as photosynthesis (BP), photosynthesis, light harvesting (BP), and photosynthesis, light harvesting in photosystem I (BP), as well as several chloroplast structural terms including photosystem (CC), photosystem I (CC), photosystem II (CC), photosystem I reaction center (CC), chloroplast thylakoid membrane (CC), and plastid thylakoid membrane (CC), were enriched exclusively with downregulated genes. This pattern is consistent with the overall downregulation observed in the KEGG pathways Photosynthesis and Photosynthesis–antenna proteins. Several stress-related processes, such as response to heat (BP) and response to temperature stimulus (BP), were enriched and contained upregulated genes. Terms associated with protein homeostasis, including protein folding (BP) and unfolded protein binding (MF), also contained upregulated genes, and pathways such as Protein processing in endoplasmic reticulum were significantly enriched in KEGG. In addition, plant–pathogen interaction and plant hormone signal transduction were prominently enriched as well.

In the DEGs between the control group and the dual-treatment group (Figure 6B,E), the GO and KEGG enrichment analyses showed that the plants exhibited transcriptional responses characterized by stress buffering, protein homeostasis maintenance, and energy metabolism remodeling. Multiple stress-related biological processes, such as response to heat (BP), response to temperature stimulus (BP), and response to abiotic stimulus (BP), were significantly enriched and contained upregulated genes. Several protein homeostasis-related GO terms, including protein folding (BP) and unfolded protein binding (MF), also featured multiple upregulated genes, and the significant enrichment of the KEGG pathway Protein processing in endoplasmic reticulum further indicated enhanced ER-associated folding and quality-control mechanisms. In addition, GO terms related to oxidoreductase activity—such as oxidoreductase activity (MF), dioxygenase activity (MF), and monooxygenase activity (MF)—were broadly enriched and upregulated. For energy metabolism, both the Fatty acid degradation and Citrate cycle (TCA cycle) pathways were fully upregulated, accompanied by the activation of several amino acid metabolic pathways. Although photosynthesis-related KEGG pathways remained dominated by downregulated genes, their suppression did not exceed that observed in the pathogen-treated group.

The GO and KEGG enrichment analyses comparing the pathogen-treated group with the dual-treatment group revealed that the biocontrol fungus markedly reshaped the plant’s molecular responses under pathogen-induced stress (Figure 6C,F). Numerous GO terms associated with protein translation and macromolecule biosynthesis, such as translation (BP), peptide biosynthetic process (BP), and cellular macromolecule biosynthetic process (BP), as well as several ribosome-related structural categories—including large ribosomal subunit (CC), ribosome (CC), ribosomal subunit (CC), ribonucleoprotein complex (CC), and protein-containing complex (CC)—were significantly enriched. Although downregulated genes remained predominant in these pathways, several ribosomal components and translation-related genes were upregulated. Multiple GO categories related to energy metabolism and cellular structure, including the inner mitochondrial membrane protein complex (CC) and mitochondrial protein-containing complex (CC), were also significantly enriched. KEGG analysis further showed that the Ribosome pathway was significantly enriched and included upregulated genes, whereas Plant–pathogen interaction and MAPK signaling pathways displayed a higher proportion of downregulated genes. Additionally, several secondary metabolism pathways, such as Phenylpropanoid biosynthesis and Sesquiterpenoid and triterpenoid biosynthesis, contained upregulated genes.

### 3.7. Mining of Candidate Genes Involved in Biocontrol-Mediated Pathogen Suppression

**Figure 7:**
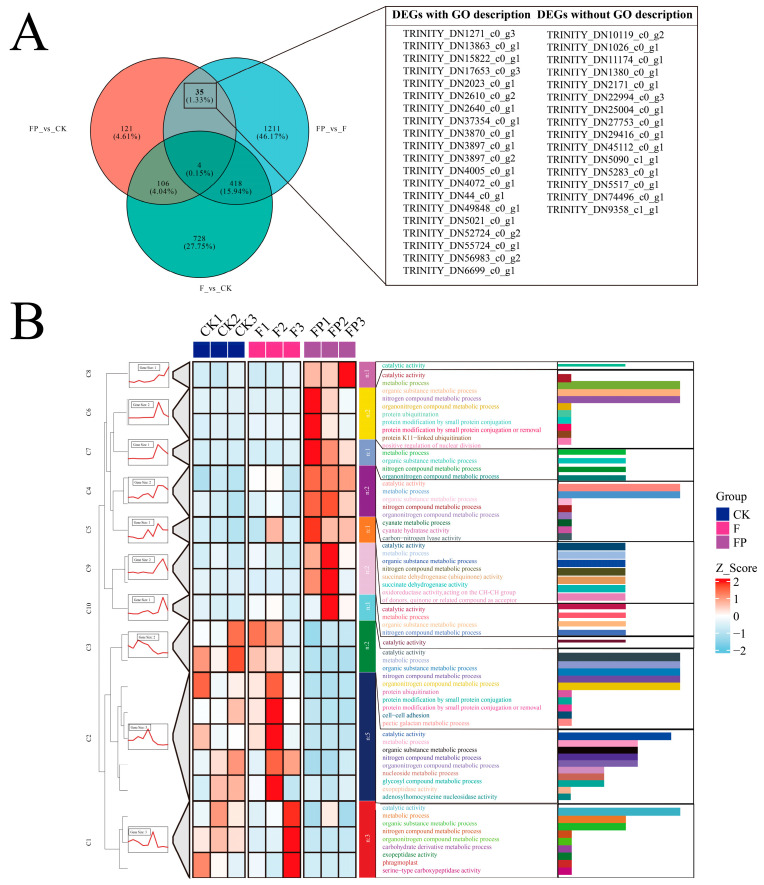
(**A**) Venn diagram of shared and unique genes among different treatment groups; (**B**) heatmap analysis of 20 DEGs. GO, Gene Ontology; DEGs, differentially expressed genes; CK, control group; F, pathogen-treated group; FP, dual-treatment group.

To explore how *P. lilacinum* BP2-7 influences the *A. membranaceus* response during its interaction with *F. solani* BN2-2, this study constructed a Venn diagram of DEGs among three treatment groups. The results showed that 35 DEGs were shared between the dual-treatment group versus the control and the dual-treatment group versus the pathogen-treated group (Figure 7A), and the number of DEGs differed significantly between the dual-treatment group and both the control and pathogen-treated groups (Figure 7B).

Among these DEGs, 20 had functional annotations. The genes highly expressed in the dual-treatment group were mainly associated with catalytic activity and various metabolic processes, including the metabolism of organic substances, nitrogen-containing compounds, and organonitrogen compounds. They also encompassed several more specific metabolic functions, such as succinate dehydrogenase activity, oxidoreductase activity, cyanate metabolic process, and carbon–nitrogen lyase activity. In addition, some genes were related to protein modifications, including protein ubiquitination and small protein conjugation as well as functions associated with cell division.

In contrast, genes that were lowly expressed in the dual-treatment group but highly expressed in both the control and pathogen-treated groups were primarily enriched in basic metabolic functions, such as catalytic activity, metabolic process, organic substance metabolic process, and nitrogen/organonitrogen compound metabolic process. These genes also included more specific annotations related to carbohydrate and nucleoside metabolism (carbohydrate derivative metabolic process, nucleoside metabolic process, glycosyl compound metabolic process), proteolytic enzyme activities (exopeptidase activity, serine-type carboxypeptidase activity), cell structure and adhesion (phragmoplast, cell–cell adhesion), and polysaccharide-related metabolism (pectic galactan metabolic process).

## 4. Discussion

### 4.1. Root Rot Drives Changes in the Microbial Community Structure Within A. membranaceus Roots and Regulates the Association Between Its Diversity and Soil Factors

Findings based on the comparison between roots showing no disease symptoms and those exhibiting root rot reveals the significant reshaping effects of root rot on the bacterial and fungal community structures within *A. membranaceus* roots. Overall, both bacterial and fungal OTU numbers were lower in diseased plants than in healthy ones. However, bacteria and fungi exhibited markedly different response patterns in terms of dominant taxon composition, diversity, and co-occurrence network structures, reflecting differences in their ecological functions and host dependencies. In the bacterial community, the ACE and Chao1 indices of diseased roots were significantly reduced, indicating that disease leads to a decline in bacterial species richness [4]. The increases in the Shannon and Simpson indices may be attributed to the disruption of dominant bacterial taxa originally present in healthy roots, which reduces their relative abundance and results in a more even community structure [34]. The changing trend of the fungal community differed from that of the bacterial community. With disease occurrence, fungal diversity decreased significantly, and the relative abundance of most dominant taxa declined, whereas genera such as *Fusarium*, *Plectosphaerella*, *Mycothermus*, and *Thanatephorus* became enriched. This pattern suggests that disease progression involves not only the proliferation of specific fungal taxa, but also an overall disruption of the microbial ecological balance in the roots [35]. Notably, although *Fusarium* was enriched in diseased roots, this genus includes not only pathogenic species, but also saprophytic, endophytic, and other non-pathogenic taxa [36]. Moreover, previous studies have reported the existence of *Fusarium*-suppressive soils [37]. Therefore, its increase is more likely to reflect disease-associated shifts in community structure rather than being taken as direct evidence of pathogenic dominance. The enrichment of other genera further suggests that selective ecological niches may be formed in diseased roots, favoring the growth of certain adapted taxa, opportunistic fungi, or secondary colonizing fungi [38]. Overall, the occurrence of root rot may drive the fungal community to shift toward lower diversity and enrichment of specific taxa [39]. Beta diversity analysis showed that the fungal community exhibited much greater dispersion across different conditions than the bacterial community, further demonstrating the high sensitivity and instability of fungi in response to disease disturbance [40]. Co-occurrence network analysis further revealed that the diseased roots had significantly higher numbers of edges and node degrees in the bacterial network compared with healthy roots [41]. In diseased roots, the fungal network exhibited fewer edges and a lower average degree compared with healthy roots, indicating weakened interactions within the fungal community. This suggests that the overall stability and functional potential of the network may be impaired [39].

This study also showed that soil physicochemical factors influence microbial diversity and richness by providing different nutritional conditions. Acidic environments and high organic matter content suppress beneficial microbes and promote the proliferation of pathogens like *Fusarium* [42]. Nutrients such as nitrogen, phosphorus, potassium, and metal elements like copper provide favorable growth conditions for bacteria and fungi [43,44]. Therefore, optimizing soil physicochemical management, particularly adjusting pH and nutrient levels, is crucial for microbial community regulation and disease control.

### 4.2. Multidimensional Control Mechanism and Disease–Growth Balance Effect of P. lilacinum BP2-7 in Suppressing A. membranaceus Root Rot

The results of both the plate confrontation assay and pot experiments showed that *P*. *lilacinum* BP2-7 significantly inhibited the mycelial growth of the root rot pathogen *F*. *solani*, and significantly reduced the disease incidence. This indicates that *P. lilacinus* has disease-suppressing potential [45]. To investigate how the biocontrol fungus influences the host response, transcriptomic analysis was conducted. Transcriptomic comparison between the control group and the pathogen-treated group showed that pathogen infection strongly suppressed photosynthesis-related processes. This observation is consistent with findings from many plant–pathogen interaction studies, in which pathogens often inhibit photosynthesis and reduce energy supply to weaken host growth, thereby facilitating their own infection and proliferation [46]. Pathogen infection simultaneously triggered typical plant stress responses, especially the significant upregulation of heat-shock-related genes. These genes are largely associated with protein folding and molecular chaperone functions [47]. These results are consistent with the GO enrichment of terms such as protein folding and unfolded protein binding observed in this study. Meanwhile, the KEGG pathway Protein processing in endoplasmic reticulum was significantly enriched, indicating that multiple genes involved in protein processing, folding, and modification underwent marked changes under pathogen treatment [48].

Transcriptomic comparison between the control group and the dual-treatment group showed that the biocontrol fungus markedly altered the plant’s response patterns under pathogen stress. The upregulation of multiple stress-related genes indicates that plants maintained or enhanced their stress responses during the dual treatment, a common physiological feature in plants facing pathogen pressure [49]. Meanwhile, the enrichment of GO terms and KEGG pathways related to protein homeostasis suggests that the dual-treatment collectively influenced the plant’s protein processing and folding processes [50]. In addition, enhanced oxidoreductase activity and related metabolic processes indicate substantial changes in the plant’s regulation of reactive oxygen species (ROS) and its maintenance of redox balance [51]. Upregulation of pathways such as fatty acid degradation and the TCA cycle reflects a trend toward energy recovery and metabolic reprogramming in plants under the influence of the biocontrol agent [52]. Taken together, the biocontrol fungus primarily modulated plant stress responses, metabolic regulation, and protein homeostasis under pathogen infection. Its overall effect tended to promote plant recovery following pathogen stress, rather than further intensifying pathogen-induced suppression or defense responses [53].

Comparison between the pathogen-treated group and the dual-treatment group revealed that the biocontrol fungus exerted a pronounced regulatory effect on plant molecular responses under pathogen-induced stress. The results showed that, relative to the pathogen-treated group, translation-related pathways and ribosomal structural categories remained predominantly downregulated in the dual-treatment group. However, several key ribosomal subunits and translation-associated genes were upregulated, indicating that the biocontrol fungus alleviated, rather than fully restored or further suppressed, the pathogen-induced inhibition of translation [54,55]. Notably, multiple terms related to metabolism and cellular structure were enriched in the dual treatment. Together with the upregulated genes in KEGG pathways such as Ribosome and Phenylpropanoid biosynthesis, this suggests that the biocontrol fungus is closely involved in metabolic reprogramming, enhancement of secondary metabolism, and maintenance of protein homeostasis under pathogen stress [56,57,58]. At the same time, the increased proportion of downregulated genes in the Plant–pathogen interaction and MAPK signaling pathways suggests that biocontrol treatment may attenuate pathogen-induced immune hyperactivation, thereby relieving plants from the heavy metabolic burden associated with sustained high-level defense responses [59,60]. Overall, combined with the transcriptomic results of this study, it can be inferred that the primary role of the biocontrol fungus following pathogen infection is not to intensify plant defenses, but rather to modulate the translation machinery, promote metabolic recovery, enhance cellular homeostasis, and mitigate immune overactivation, helping plants transition from a state of severe stress toward recovery [35,61].

### 4.3. Functional Characteristics and Multi-Pathway Coordinated Regulatory Mechanisms of Candidate Genes Involved in Biocontrol-Mediated Disease Suppression

Among the candidate genes associated with biocontrol-mediated disease suppression, 20 functionally annotated genes exhibited significant functional heterogeneity, suggesting that the disease-suppressive mechanisms of the biocontrol fungus are complex and rely on the coordinated action of multiple plant structures and functional pathways [62]. In this study, the gene expression patterns of the dual-treatment group exhibited pronounced features of metabolic reprogramming. The highly expressed genes in the dual-treatment group were primarily enriched in catalytic activities and various metabolic processes, particularly those involving organic compounds, nitrogen-containing compounds, and their derivatives. These genes also included functions closely related to energy supply and redox regulation, consistent with the findings of Wang Y.P. et al. [63]. Additionally, some genes were associated with protein ubiquitination, small-molecule conjugation, and cell division, suggesting that the dual treatment not only enhanced basal metabolic activity but may also regulate protein homeostasis and cellular turnover to adapt to combined biotic stimuli [49,64].

In contrast, genes highly expressed in the control group and the pathogen-treated group were mostly related to fundamental growth and metabolic processes, including carbohydrate and nucleotide metabolism, protease activity, cellular structural and adhesion functions, and polysaccharide-related pathways. This expression pattern suggests that the dual treatment may shift resource allocation away from routine growth and energy-intensive processes such as cell wall remodeling, redirecting it toward stress responses and metabolic regulation to maintain metabolic balance and adaptability under complex stress conditions [65].

Taken together, these 20 DEGs likely reflect host defense responses induced by *P. lilacinum*. Therefore, the DEGs identified in this study represent key defensive molecular responses employed by the host to mitigate pathogen damage, providing new evidence for the indirect biocontrol mechanisms of *P. lilacinum*.

## 5. Conclusions

Root rot caused by *F. solani* significantly reshaped the endophytic microbial community structure of *A. membranaceus*, with the fungal community showing a more pronounced response to disease than the bacterial community. The biocontrol fungus *P. lilacinum* BP2-7 exhibited strong inhibitory activity against *F. solani* in both dual-culture and pot experiments, indicating considerable potential for the biological control of *A. membranaceus* root rot. Transcriptomic analysis further revealed that the effect of *P. lilacinum* BP2-7 was not simply to enhance host defense, but rather to alleviate pathogen-induced excessive immune responses by regulating translation, promoting metabolic reprogramming, and maintaining cellular homeostasis, thereby helping diseased plants gradually transition from a damaged state toward recovery. In addition, this study identified 20 candidate genes that may be involved in the direct inhibition of *F. solani*, providing new clues for elucidating the antifungal mechanism of *P. lilacinum* BP2-7. Overall, this study not only reveals the ecological impact of root rot on the root microbial community of *A. membranaceus*, but also clarifies the molecular basis of *P. lilacinum* BP2-7-mediated disease suppression, thus providing a theoretical foundation for the development of green control strategies against soil-borne diseases.

## Data Availability

The original data presented in the study are openly available in NCBI Sequence Read Archive (SRA) at accession number PRJNA 1379149.

## Appendix A

**Table A1:** PCR reaction system.

Added Reagents	Added/μL
2*Taq Plus PCR Master Mix	10
Primer	0.5
Primer	0.5
Template DNA	3
ddH_2_O	6

**Table A2:** PCR reaction conditions.

Temperature	Time	Cycles
94 °C (initial denaturation)	4 min	-
94 °C (denaturation)	30 s	30 cycles
50 °C (annealing)	30 s	30 cycles
72 °C (extension)	1 min 40 s	30 cycles
72 °C (final extension)	10 min	-

**Table A3:** Grading standard of root rot disease of *Astragalus membranaceus*.

Rank	Trait
Level 0	Plant roots are normal, the whole plant is healthy.
Level 1	1–2 leaves of the plant are wilted.
Level 2	3–5 leaves of the plant are wilted.
Level 3	6–8 leaves of the plant are wilted.
Level 4	9–10 leaves of the plant are wilted.
Level 5	The whole plant is dead.

**Table A4:** Disease assessment of *A. membranaceus* under different treatments.

Treatment		F	FP
Disease incidence (%)	Level 0	0	0
	Level 1	5	10
	Level 2	1	1
	Level 3	20	0
	Level 4	15	0
	Level 5	40	0
Disease severity index (%)		69	25
